# Can nebulised heparin reduce acute lung injury in patients with SARS‑CoV‑2 requiring advanced respiratory support in Ireland: the CHARTER‑Ireland phase Ib/IIa, randomised, parallel-group, open-label study

**DOI:** 10.1186/s40635-025-00727-x

**Published:** 2025-02-07

**Authors:** David Cosgrave, Bairbre McNicholas, Ciara Hanley, John Robert Sheehan, Padraig Calpin, Maeve Kernan, Darragh Murphy, Alberto Alvarez‑Iglesias, John Ferguson, Camilla Giacomini, Christine Greene, Catriona Cody, Shane McGeary, Marion Murphy, Marianne Fitzgerald, Gerard Curley, Barry Dixon, Roger J. Smith, Claire Masterson, Daniel O’Toole, Frank van Haren, John G. Laffey

**Affiliations:** 1 Department of Anaesthesia and Intensive Care Medicine, University Hospital Galway, Galway, Ireland; 2 Anaesthesia and Intensive Care Medicine, School of Medicine, University of Galway, Galway, Ireland; 3 HRB Clinical Research Facility, School of Medicine, University of Galway, Galway, Ireland; 4 School of Mathematical & Statistical Sciences, University of Galway, Galway, Ireland; 5 Department of Anaesthesia and Intensive Care Medicine, Connolly Memorial Hospital Blanchardstown, Dublin, Ireland; 6 Department of Anaesthesia and Intensive Care Medicine, Limerick University Hospital, Limerick, Ireland; 7 Department of Anaesthesia and Intensive Care Medicine, Royal College of Surgeons in Ireland, Beaumont Hospital, Dublin, Ireland; 8 Department of Critical Care Medicine, St Vincent’s Hospital Melbourne, Fitzroy, VIC Australia; 9 Intensive Care Unit, St George Hospital, Sydney, Australia; 10 College of Health and Medicine, Australian National University, Canberra, Australia

**Keywords:** Heparin, Nebulised, Aerosol delivery, COVID-19, Acute respiratory distress syndrome, Safety study

## Abstract

**Background:**

Nebulised unfractionated heparin may attenuate COVID-19 ARDS by reducing pulmonary microvascular thrombosis, blocking SARS-CoV-2 entry into cells, and decreasing lung inflammation. COVID-19 patients with a raised d-dimer have areas of pulmonary hypoperfusion on CT perfusion scans of the lung and have increased mortality risk.

**Methods:**

This was a phase Ib/IIa open-label multi-centre, randomised controlled trial. The study was designed to evaluate whether nebulised unfractionated heparin decreased d-dimer concentrations, with safety as a co-primary outcome.

**Results:**

Forty patients were recruited, with 20 patients into each group. Mean age was 56.6 (SD 11.5) in the heparin group and 51.3 (SD 14.7) in the standard care group, while 60% of participants were male. There was no change in d-dimers from baseline to day 10 (heparin group mean change − 316.5, [SD 1840.3] and control group mean change − 321.7 [SD 3589.4]; *p* = 0.996). Fourteen patients suffered at least one serious adverse event, 9 patients the Heparin group and 5 in the control group. Eight patients had one or more bleeding events, 5 in the heparin group and 3 in the control group, but were no cases of pulmonary bleeding, of severe haemorrhage or of heparin-induced thrombocytopenia. Patients receiving heparin therapy had lower PaO_2_/FiO_2_ ratios, increased oxygenation indices, and decreased ROX index profiles, up to day 10. The time to separation from respiratory support, and the time to ICU or hospital discharge was similar in both groups. There were 3 deaths in the Heparin group and 2 in the control group.

**Conclusions:**

Nebulised unfractionated heparin was safe and well tolerated, but did not reduce d-dimer concentrations, and worsened oxygenation indices in patients with COVID-19 ARDS.

**Supplementary Information:**

The online version contains supplementary material available at 10.1186/s40635-025-00727-x.

## Background

Clinical studies of nebulised heparin in patients with acute respiratory distress syndrome (ARDS) have shown it to be a safe intervention [1] and have shown promising results in reduction of progression of lung injury and earlier hospital discharge [2]. The COVID-19 pandemic has resulted in a high volume of patients presenting to critical care with ARDS [3]. The pathophysiology of ARDS seen includes a markedly raised d-dimer level, indicative of a hypercoagulable state [3–6]. Previous studies have shown microvascular thrombosis as a distinct clinical feature of ARDS, leading to hyaline membrane formation and fibrosis [1]. Furthermore, patients with a raised d-dimer have areas of hypoperfusion on lung CT perfusion scans. These patients have a markedly increased mortality compared to patients with d-dimer s less than the median value of enrolled patients in these studies [3].

Heparin can alter the conformation of the SARS-CoV-2 spike protein in vitro, reducing viral entry into cells [7]. Heparin also has anti-inflammatory effects, where a reduction in pro-inflammatory markers in an ARDS cellular model has been demonstrated [8]. Consequently, nebulised heparin may have therapeutic potential for COVID 19 ARDS [9]. The CHARTER Ireland Study is a preliminary safety and efficacy study, with a co-primary outcomes of safety and d-dimer concentrations [10]. The study aims to test the hypothesis that nebulised unfractionated heparin will reduce microthrombus formation in the lung microvasculature, as evidenced by a reduction in the surrogate marker of d-dimers.

## Methods

This phase Ib/IIa study is an investigator initiated, prospective randomised, parallel-group, multi-centre open label, proof of principle, superiority study. The study took place across three intensive care units (ICUs) in Ireland. As a phase Ib/IIa study, the co-primary endpoints were safety outcomes and efficacy as evidenced by changes in the d-dimer profile in recruited patients.

The study was registered with the EU clinical trials database (2020-003349-12) and clinicaltrials.gov (NCT04511923), registered 11/08/2020. Ethics approval was obtained from the National Research Ethics Committee (NREC) in Ireland, approval number 20-NREC-COV-104. Regulatory approval for the study was obtained from the Health Products Regulatory Authority, approval number CT0900/650/001 Heparin Sodium. The protocol and statistical analysis plan for this study has been peer reviewed and published previously [10].

### Eligibility criteria

Inclusion criteria for this study were as follows: (1) confirmed or suspected COVID-19 (if ‘suspected’, results must be pending or testing intended); (2) ability to obtain informed consent/assent to participate in study; (3) age 18 years or older; (4) requiring high-flow nasal oxygen (> 30L/min) or positive pressure ventilator support or invasive mechanical ventilation in the ICU for a time period of no greater than 48 h; (5) d-dimers > 200 ng/ml; (6) acute opacities (not effusions, lobar/lung collapse or nodules) on chest imaging affecting at least one lung quadrant; (7) currently in a higher level of care area designated for inpatient care of patients where advanced respiratory support therapies can be provided.

Exclusion criteria included the following: Enrolled in another clinical trial that was unapproved for co-enrolment, heparin allergy or heparin-induced thrombocytopenia, APTT > 100 s, pulmonary bleeding, platelet count < 50 × 10^9^ per L, uncontrolled bleeding, pregnancy, technical/equipment constraints, clinician objection, the use or anticipated use of nebulised tobramycin during this clinical episode, any other specific contraindication to anticoagulation, receiving any direct/novel oral anticoagulant. The full exclusion criteria are described in detail in the methods paper [10].

### Conduct of the study

Patients meeting the eligibility criteria were approached for consent to participate, or in the case of patients incapable of providing consent, deferred consent and relative assent to participate were sought. The process was approved by the Health Research Consent Declaration Committee in Ireland.

Allocation was carried out after consent or assent were confirmed, via a central, secure web randomisation process embedded in the eCRF. The eCRF was hosted by the contract research organisation (CRO, Afortiori Development, Galway, Ireland). Allocation was in a one-to-one ratio, with variable block size randomisation. The Programme R was used to generate the randomisation sequence. Blocks of variable size and a random seed were used to ensure allocation concealment. Block size was not revealed to site investigators by the CRO. Site-level randomisation was used. Participants were assigned to receive standard ICU care plus nebulised heparin or standard care alone.

In the treatment group, nebulised unfractionated heparin 25,000 units (5 ml heparin sodium 5000 IU/ml (Pinewood laboratories, Clonmel, Ireland) was administered via the Aerogen Solo® nebuliser every 6 h from enrolment to day 10, or until discontinuation of advanced respiratory support (if sooner), with the dosage and schedule based on previous work [1, 11, 12]. The control standard of care (SOC) group received standard care.

### Study endpoints

Outcomes are based on data collected up to day 10 for laboratory and some clinical results, with other clinical endpoints and safety and adverse event data collected up to day 60. All outcomes and timepoints are detailed in the methods paper [10].

*Primary outcomes.* The co-primary outcomes were a between-group difference in d-dimer concentrations over time, and a between-group difference in the occurrence of serious adverse events (SAEs) as listed in the safety analysis.

*Secondary outcomes.* Secondary efficacy outcomes include analysis of between-group differences in: P/F ratio and pulmonary compliance (in invasively ventilated patients). The ROX index, defined as the ratio of oxygen saturation as measured by pulse oximetry/F_I_O_2_ to respiratory rate was assessed in patients receiving high-flow nasal oxygen (HFNO) therapy. The oxygenation index (OI = mean airway pressure MAP × FiO_2_ × 100 ÷ PaO_2_) was assessed in patients receiving positive pressure ventilation. The time to separation from invasive ventilation to day 28, number tracheotomised to day 28 (N), time to ICU discharge, and survival (day 28, day 60 and hospital discharge) were also assessed. Inflammatory and coagulation indices included interleukins (IL-1β, IL-6, IL-8, IL-10), soluble tumour necrosis factor receptor 1, C-reactive protein, procalcitonin, ferritin, fibrinogen and lactate dehydrogenase), ratio of IL-1β/IL-10 and IL-6/IL-10.

*Safety outcomes.* These include analysis of between-group differences in: patients transfused packed red blood cells (PRBCs); volume of PRBCs transfused; major bleeding, (defined as bleeding that results in death and/or bleeding that is symptomatic and occurs in a critical area or organ); bleeding that results in a fall in haemoglobin of 20 g/l or more, or results in transfusion of > 2 units of PRBCs; number of patients who record clinically relevant non-major bleeding as defined by the International Society on Thrombosis and Haemostasis [13], number developing heparin-induced thrombocytopenia (HIT), number of other adverse events and reactions, all to day 10. AEs were classified by organ system. Safety data on air quality samples from an infection control perspective will be published separately.

*Sample size.* As a phase Ib/IIa study, and as published data relating to COVID 19 on which to power this study were lacking at that time, we based our sample size calculation on in-house data for d-dimer levels in COVID-19 patients who required ICU care (12 patients) and those who did not (26 patients). Based on an estimation that nebulised heparin may reduce the d-dimer levels in ICU patients (mean = 944.8 ng/ml [SD = 485.3]) to those experienced in ward patients (mean = 436.5 ng/ml [SD = 604.0]), with an alpha level of 0.05 and a power of 90% to detect a type II error, 19 patients per group were required. Increasing the number by 1 per group to allow for potential dropout gave a sample size of 20 patients per arm of the study, with a total of 40 patients to be enrolled. The safety analysis was not used to power the study, but the safety outcomes were analysed as a co-primary outcome.

### Statistical analysis

Demographic data were summarised appropriately by treatment arm. The primary outcomes analysis was carried out on an intention to treat basis. Safety analysis was carried out also, including all recruited patients.

Effectiveness analysis for the primary outcome was carried out using analysis of covariance for d-dimer AUC, adjusting for baseline AUC, and Fisher’s exact test for safety outcomes. Continuous outcomes were statistically compared between treatment groups using t-tests or Mann–Whitney *U* tests (for non-normal variables). Linear mixed effects models, were used for the analysis of longitudinal data, representing the effect of heparin by differential slopes of response trajectories over time between the heparin and control arms. Responses were log-transformed, prior to fitting these models, when distributions were notably skewed. A Cox proportional hazards model was used to model time to event responses. Interval estimates (95% confidence intervals) for the treatment effect were reported in addition to the point estimates, for all response variables [10].

## Results

**Fig. 1:**
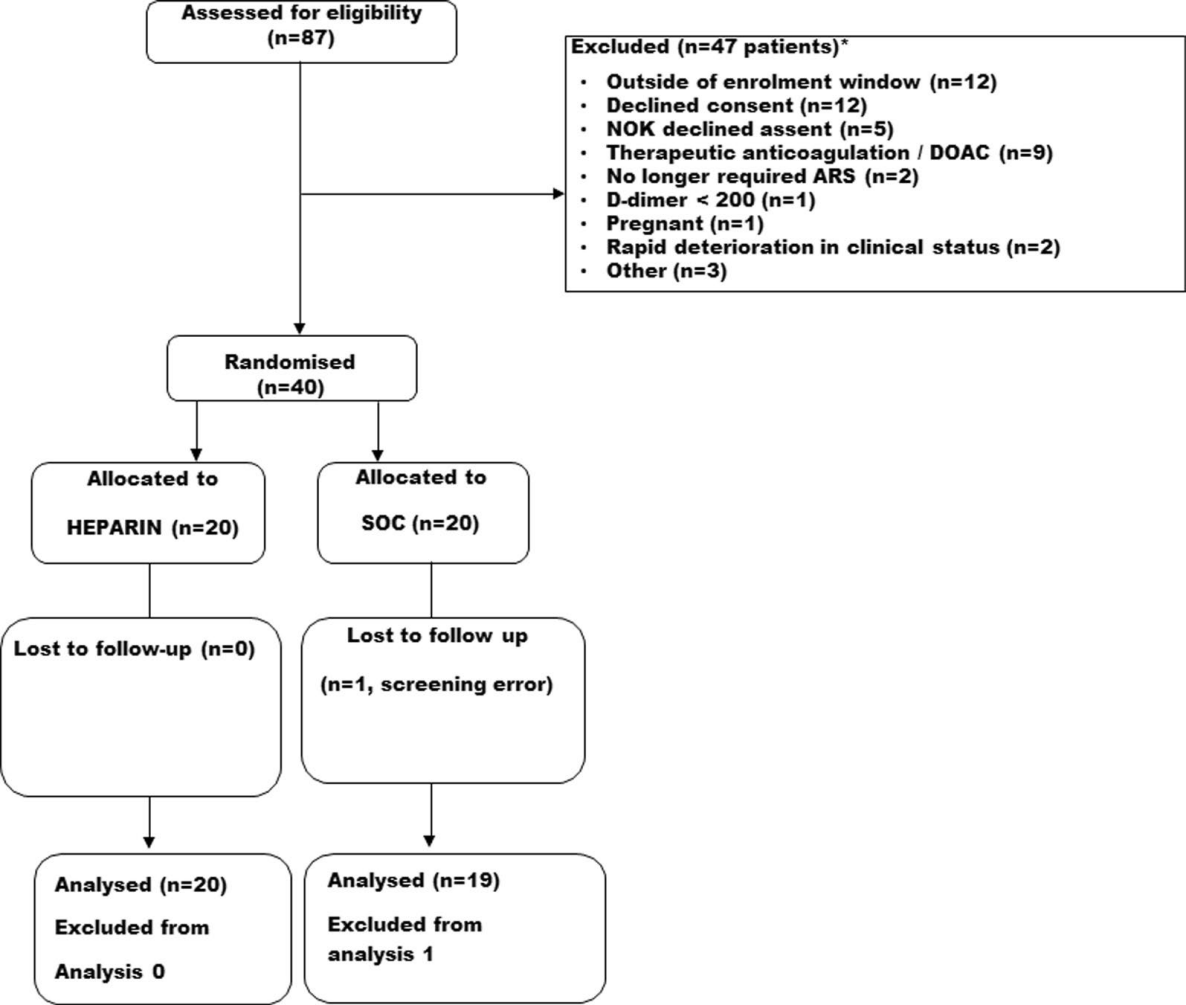
Consort flowchart for CHARTER-IRELAND Study

From 1st December 2020 to 28th December 2021, 87 patients were screened for eligibility, of whom 47 were excluded (Fig. 1—CONSORT). 40 patients were recruited with 20 allocated to each group (Fig. 1). One patient was subsequently completely withdrawn from the study due to a screening error. The primary outcome was therefore available for 39 patients (20 in Heparin group and 19 in SOC group).

Baseline patient characteristics and supportive care were similar between the groups (Table 1), and patients were typical of a critically ill population with COVID-19 [14], with predominant respiratory failure indicated by the low PF ratio and high OI, and comparatively low SOFA score. Vaccination status, corticosteroid and antiviral use were similar in both groups (Table 1).

**Table 1:** Baseline patient characteristics

	Heparin treatment [*N* = 20]	Standard care [*N* = 19]
Patient characteristics		
Age, years; mean (SD)	56.6 (11.5)	53.1 (12.9)
Sex, male; *n* (%)	13 (65%)	11 (57.9%)
BMI; (mean, SD) (N 19,16)	31.1 (5.2)	32.6 (5.7)
Ethnicity; *n* (%)		
Caucasian	19 (95%)	17 (89.5%)
Black, African, Caribbean or Ethnic Black	0 (0%)	2 (10.5%)
Asian or Ethnic Asian	1 (5%)	0 (0%)
COVID-19 diagnosis; *n* (%)		
Confirmed	19 (95%)	19 (100%)
Suspected	1 (5%)	0 (0%)
Concomitant/prior medications		
Vaccinated against COVID-19; n (%)	7 (35%)	9 (47.4%)
Antiviral medications (e.g. remdesivir); *n* (%) (N 19,19)	1 (5.3%)	0 (0%)
Steroids (e.g. dexamethasone); *n* (%) (N 19,19)	16 (84.2%)	18 (94.7%)
Other immunomodulatory drugs (e.g. anti-IL-6); *n* (%) (N 19,19)	3 (15.8%)	4 (21.1%)
Baseline severity indices		
First qualifying PaO_2_/FiO_2_ ratio; mean (SD)	179.6 (64.2)	134.9 (45.1)
Worst PaO_2_/FiO_2_ ratio (first 24 h); mean (SD) (N 19,19)	158.4 (63.2)	148.5 (59.7)
Total SOFA score; median (IQR)	3 (5)	4 (6)
Lowest mean arterial pressure (mmHg); mean (SD)	88.5 (15)	84.5 (15.4)
Vasopressor infusion treatment; *n* (%)	3 (15%)	6 (31.6%)
Ferritin (μg/L); median (IQR) (N 12,11)	1586.5 (2226)	1239 (690.5)
C-reactive protein (mg/L); median (IQR) (N 20,19)	65.6 (41.2)	43.2 (70.9)
Procalcitonin (ng/ml); median (IQR) (N 18,18)	0.1 (0.1)	0.1 (0.2)
Baseline respiratory support parameters		
Type of respiratory support; *n* (%)		
High-flow nasal oxygen	10 (50%)	8 (42.1%)
CPAP/non-invasive ventilation	3 (15%)	5 (25%)
Invasive mechanical ventilation	7 (35%)	6 (31.6%)
Oxygenation index; median (IQR) (N 10,10)	71.5 (375.9)	107.4 (229.6)
Dynamic pulmonary compliance (mLs/cmH2O); mean (SD) (N 7,7)	36.6 (16.6)	44.8 (22.9)
Adjunctive therapies		
Neuromuscular blocking drugs; *n* (%)	6 (30%)	7 (36.8%)
Prone position; *n* (%)	12 (60%)	9 (47.4%)

Interquartile range (IQR) is presented as the difference between quartile 1 and quartile 3

### Safety outcomes

The overall incidence of adverse events was similar in both groups. There were 21 serious adverse events in total in the study, with 14 patients suffering at least one serious adverse event, of whom 9 were the Heparin group and 5 in the SOC group; risk ratio RR = 1.8 (CI 0.73 to 4.43) *p* = 0.208 (Table 2). Eight patients suffered haemorrhages during the trial, 5 in the Heparin arm and 3 in the SOC group (Table 2). These haemorrhages were all graded mild or moderate in severity and there were no severe haemorrhages, or pulmonary haemorrhages in either group. No blood product transfusions were recorded. No serious adverse events were reported related to the nebulised Heparin. A list of all serious adverse events (Table E1) and detailed listing of all bleeding-related adverse events reported (Table E2) are provided in the online supplement.

**Table 2:** Safety outcomes

	Heparin treatment [N = 20]	Standard care [N = 19]	Relative risk (95% confidence intervals)	*p*-value
Patients with adverse events at any stage; *n* (%)	16 (80%)	14 (73.7%)	RR = 1.09 (CI 0.77 to 1.54)	0.663
Patients with serious adverse events at any stage; *n* (%)	9 (45%)	5 (26.3%)	RR = 1.71 (CI 0.7 to 4.18)	0.249
Patients with haemorrhage at any stage; *n* (%)	5 (25%)	3 (15.8%)	RR = 1.58 (CI 0.44 to 5.73)	0.512
Total number haemorrhages; *n*	13	9		
Haemorrhage severity; *n*				
Mild	9	2		
Moderate	4	7		
Severe	0	0		
Patients requiring blood transfusion; *n* (%)	0 (0%)	0 (0%)		
Patients with thromboembolic events; *n* (%)	1 (5%)	1 (5.3%)	RR = 0.95 (CI 0.06 to 14.13)	0.974

Nebulised heparin administration was well-tolerated with no significant difference between the groups in post-nebulisation (up to 1 h) haemodynamic, or respiratory indices (Table E3).

### Primary efficacy outcome

**Fig. 2:**
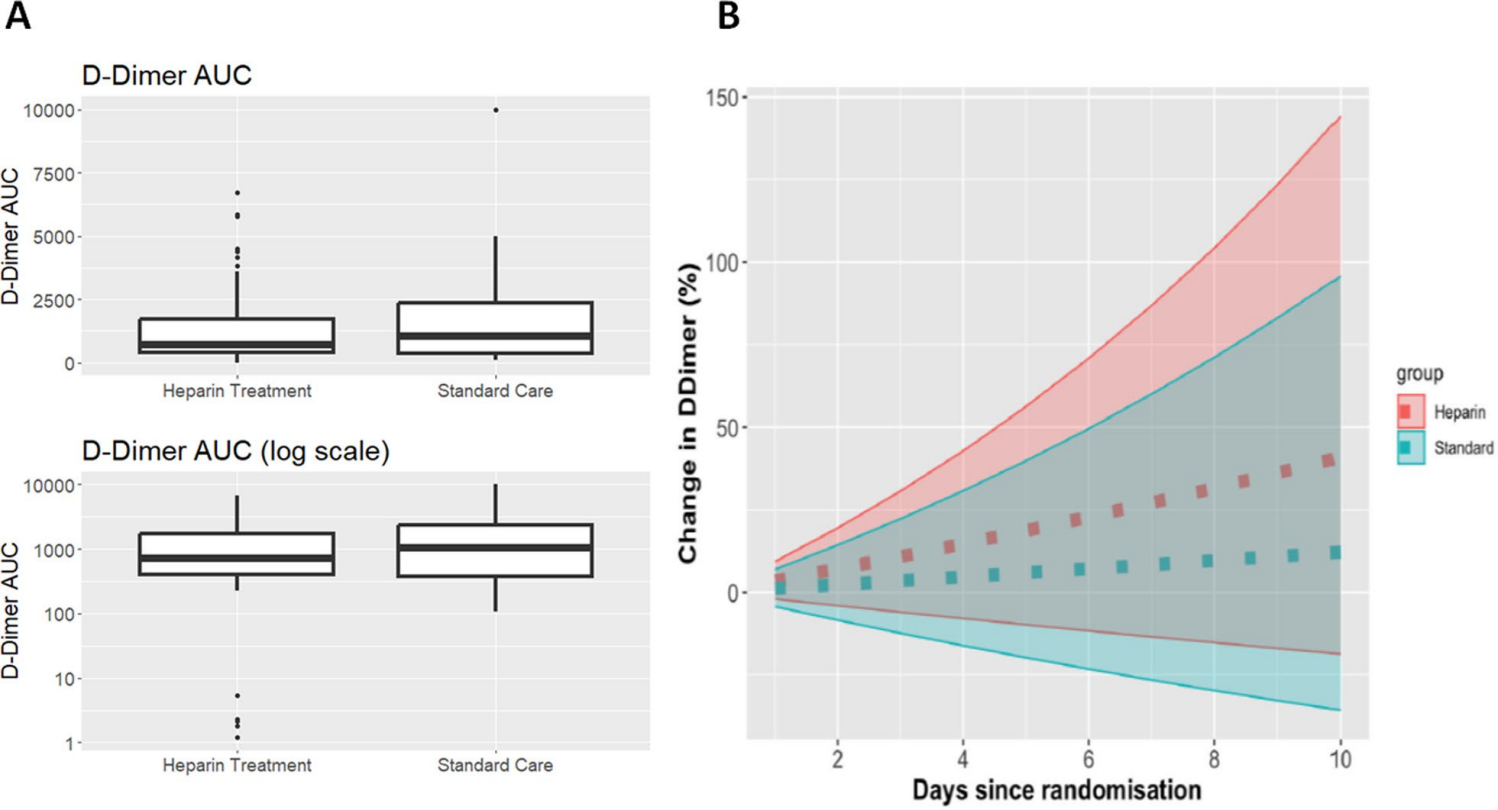
d-Dimer profile in heparin and standard of care (SOC) groups over time. **A** Boxplot of the area under the d-dimer curve in all patients receiving heparin treatment and SOC. **B** Change in d-dimer concentration over time from baseline to day 10 in all patients receiving heparin treatment and SOC

There was no difference in the primary unadjusted analysis for change in d-dimers at day 10 using imputed data from the last available data carried forward (heparin mean − 316.5, SD [1840.3] and SOC group (mean − 321.7 [SD 3589.4]; mean difference 5.2; 95% confidence interval − 2234.8 to 2245.1; *p* = 0.996) (Fig. 2A). In addition, there was no between-group difference in the area under the time curve for d-dimer concentrations (Fig. 2B) (*p* = 0.61).

### Secondary clinical outcomes

**Fig. 3:**
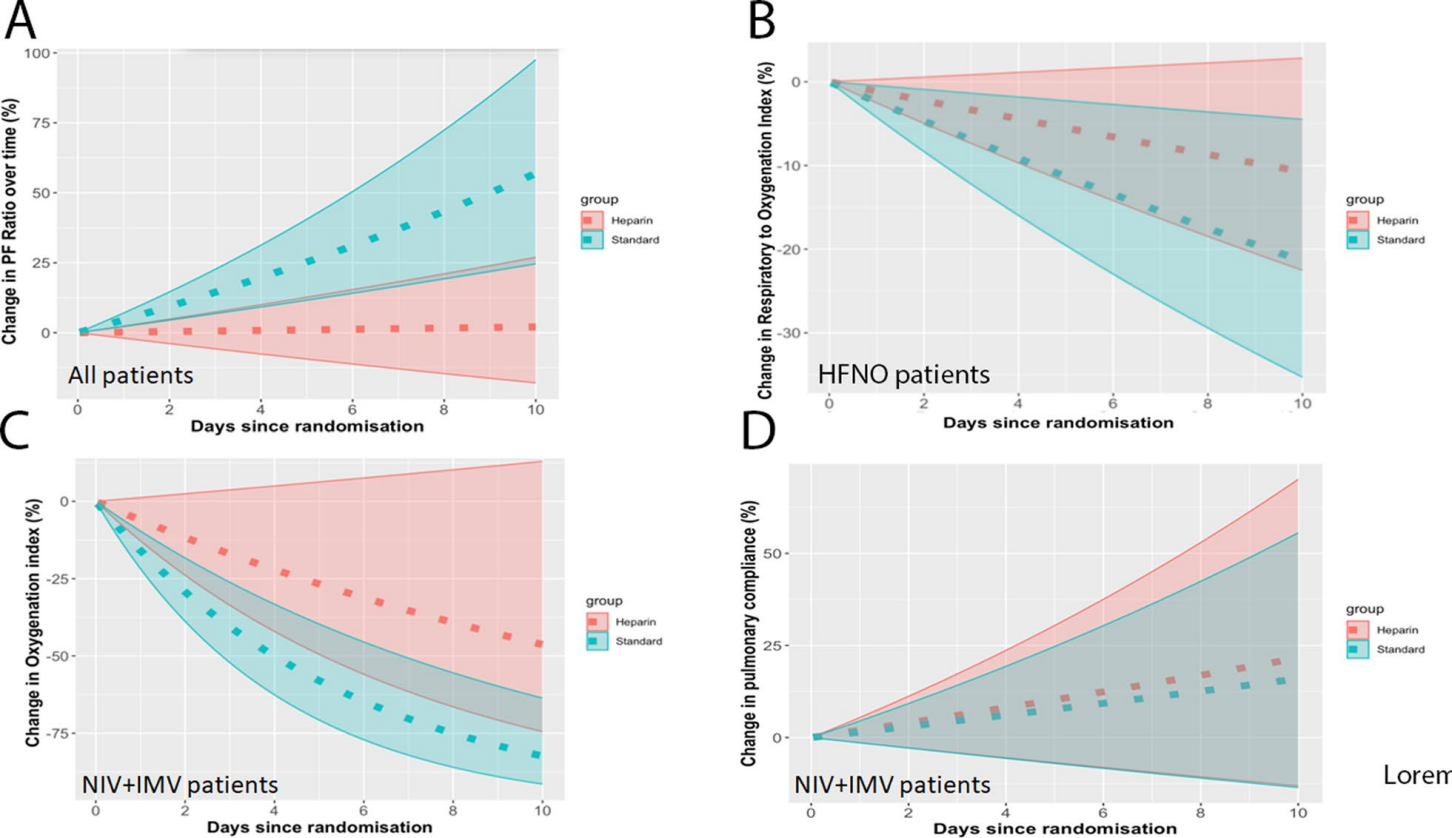
Evolution of respiratory indices from baseline to day 10. **A** Evolution of PaO_2_/FiO_2_ ratio in all patients receiving heparin treatment and SOC. **B** Evolution of ROX index in high flow nasal oxygen therapy patients receiving heparin treatment and SOC. **C** Evolution of oxygenation index in heparin and SOC patients receiving invasive and non-invasive mechanical ventilation. **D** Evolution of pulmonary compliance in heparin and SOC patients receiving invasive and non-invasive mechanical ventilation

Respiratory indices improved over time from baseline to day 10 in both the heparin and the SOC groups, but the rate and degree of improvement was significantly greater in the SOC group (Figs. 3 and e1, Table 3). The increase in PaO_2_/FiO_2_ ratio over time was greater in the SOC group, in all patients (*p* = 0.002, Fig. 3A, Table 3) and in patients receiving invasive or non-invasive MV (*p* = 0.004, Figure e1A). The decrease in ROX index over time was greater in the SOC group in patients receiving HFNO therapy (*p* = 0.002, Fig. 3B). The decrease in oxygenation index over time was greater in the SOC group, in patients receiving invasive or non-invasive MV (*p* = 0.03, Fig. 3C, Table 3), and in the subgroup of patients receiving invasive MV (Figure e1C). In contrast, there was no difference in lung compliance profiles in patients receiving invasive or non-invasive MV (*p* = 0.8, Fig. 3D), when compared to patients receiving SOC.

**Table 3:** Secondary clinical outcomes

	Heparin treatment [*N* = 20]	Standard care [*N* = 19]	Differences, hazard ratio HR or relative risk RR (95% confidence intervals)	*p*-value
P/F ratio delta (final–baseline) median (IQR)	7 (91)	74 (77)	− 67 (− 116 to − 18)	0.008
ROX index delta (final–baseline) median (IQR)	− 0.2 (1.24)	− 0.5 (1.55)	0.3 (− 0.66 to 1.09)	0.588
Oxygenation index delta (final–baseline) median (IQR)	− 2 (29)	− 9 (48)	8 (− 5 to 40)	0.113
Lung compliance delta (final–baseline) (N 7,6) median (IQR)	5 (22)	5 (10)	9.5 (− 10.9 to 47.6)	0.534
Number of patients requiring Tracheostomy to day 28 *n* (%)	1 (5%)	2 (11%)	− 5.5% (− 27% to 16%)	0.963
Time to separation from respiratory support (days) median (IQR)	9 (24)	5 (26)	HR = 0.9 (0.47 to 1.7)	0.742
Length of ICU stay (days) median (IQR)	10.5 (8.65)	7.8 (22.8)	HR = 0.99 (0.5 to 1.99)	0.987
Length of hospital stay (days) median (IQR)	22.8 (31.9)	21.6 (34.6)	HR = 1.01 (0.48 to 2.13)	0.974
28-day mortality; *n* (%)	1 (5%)	1 (5.3%)	RR = 0.95 (CI 0.06 to 14.13)	0.974
60-day mortality; *n* (%)	3 (15%)	2 (10.5%)	RR = 1.43 (CI 0.27 to 7.61)	0.712

Interquartile range (IQR) is presented as the difference between quartile 1 and quartile 3

**Fig. 4:**
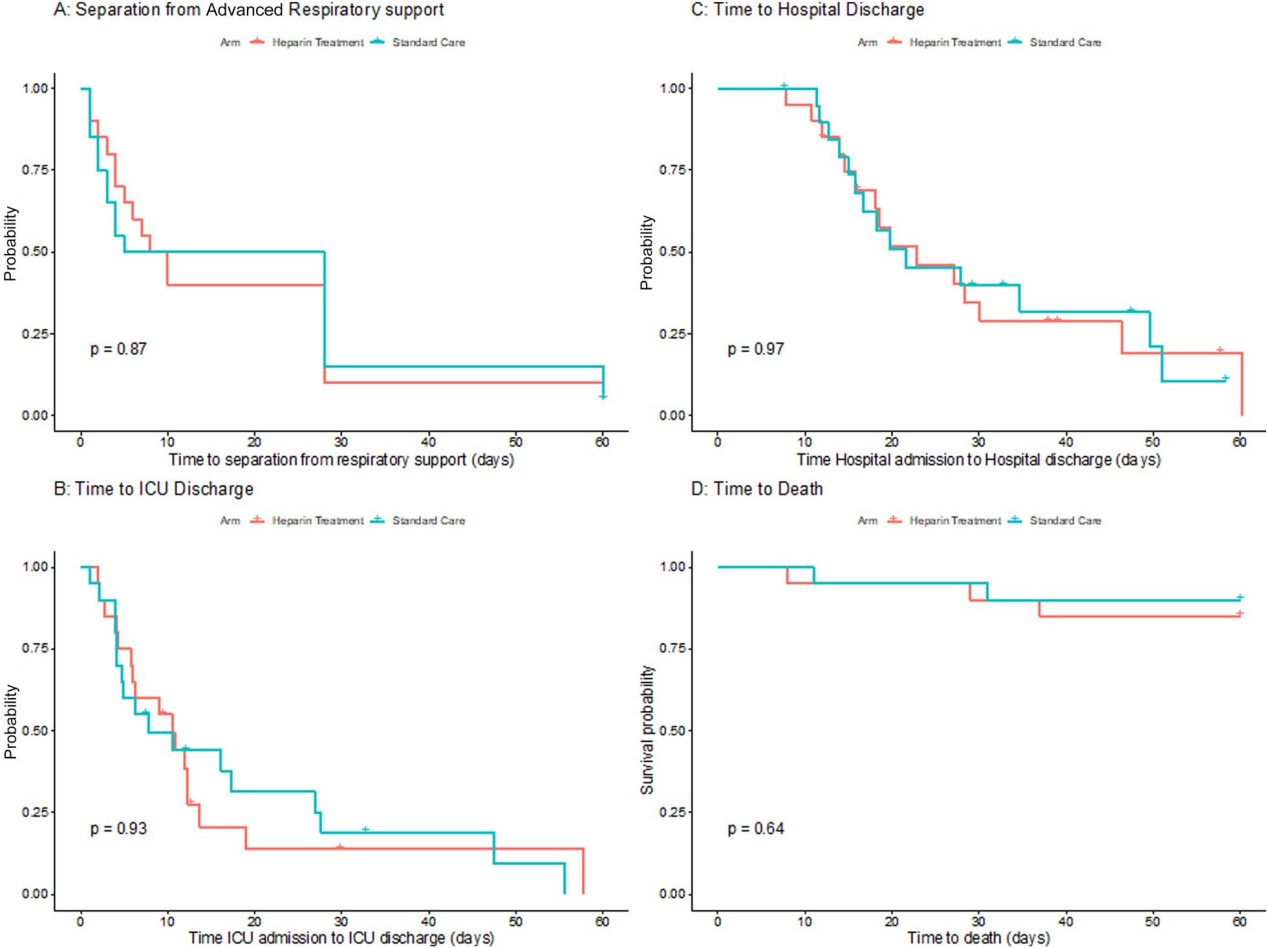
Kaplan–Meier analysis of outcome indices. **A** Time to separation from advanced respiratory support in all patients receiving heparin treatment and SOC. **B** Time to ICU discharge in all patients receiving heparin treatment and SOC. **C** Time to hospital discharge in all patients receiving heparin treatment and SOC. **D** Time to death in all patients receiving heparin treatment and SOC

There was no effect of heparin therapy on key clinical outcomes (Table 3). Specifically, the time to separation from advanced respiratory support (*p* = 0.6, Fig. 4A, Table 3). There was no effect of heparin on the time to ICU discharge (Fig. 4B, Table 3), or the time to hospital discharge (Fig. 4C, Table 3). There were 3 deaths in the heparin group and 2 in the SOC group, with no effect of heparin therapy on the time to death (Fig. 4B, Table 3). Of the patients not received invasive ventilation at study enrolment, 5 patients subsequently received invasive ventilation, of which 2/13 (15%) were in the heparin group and 3/13 (23%) in the SOC group.

### Laboratory analysis

There was no between-group differences in indices of infection or inflammation at baseline or over time to day 10 (Table 4). Specifically, concentration profiles of Interleukin 1, interleukin 6, soluble TNFa, interleukin 10, C-reactive protein and procalcitonin were not different between the groups. There was no between-group differences in indices of coagulation (prothrombin time, activated partial thromboplastin time, and fibrinogen) at baseline or over time to day 10 (Table e4).

**Table 4:** Indices of infection and inflammation over time

	Heparin treatment [*N* = 20]	Standard care [*N* = 19]	*p*-value
Interleukin 1β mean (SD)			
Baseline (N 14, 18)	2.9 (1.6)	2.6 (1.7)	0.569
Final (N 16, 15)	2 (1.1)	2.6 (1.5)	0.256
Delta (final–baseline) (N 13, 14)	− 0.8 (1.9)	− 0.1 (1.3)	0.295
Interleukin 6 median (IQR)			
Baseline (N 17, 18)	32 (91)	87 (223.8)	0.096
Final (N 19, 17)	91 (216.1)	171 (296)	0.616
Delta (final–baseline) (N 17, 16)	31 (123)	36 (336.8)	0.958
Interleukin 10 median (IQR)			
Baseline (N 12, 15)	5.5 (5.3)	2.1 (3.1)	0.213
Final (N 14, 13)	1.2 (6.2)	1.6 (2.4)	0.942
Delta (final–baseline) (N 10, 12)	− 1.8 (7.8)	− 0.7 (6.4)	0.923
Soluble TNF R1 median (IQR)			
Baseline (N 14, 18)	691.2 (347.2)	995.2 (681.4)	0.084
Final (N 17, 15)	1062.6 (796.2)	690.7 (625.8)	0.123
Delta (final–baseline) (N 13, 14)	474 (1291.7)	− 129.2 (1191.6)	0.169
C-reactive protein median (IQR)			
Baseline (N 20, 18)	65.6 (41.2)	43.2 (70.9)	0.349
Final (N 20, 17)	3.9 (17.3)	2.8 (18.7)	0.483
Delta (final–baseline) (N 20, 16)	− 63.8 (56)	− 36.4 (108.2)	0.814
Procalcitonin median (IQR)			
Baseline (N 18, 18)	0.1 (0.1)	0.1 (0.2)	0.568
Final (N 20, 16)	0.1 (0.1)	0.1 (0.1)	1
Delta (final–baseline) (N 18, 15)	0 (0.1)	− 0.1 (0.6)	0.337

Interquartile range (IQR) is presented as the difference between quartile 1 and quartile 3

## Discussion

In this phase Ib/IIa clinical trial, we found that nebulised unfractionated heparin was safe and well tolerated in patients with severe COVID-19 induced respiratory failure requiring advanced respiratory support. However, we found no evidence to suggest that nebulised heparin was effective. There was no effect on d-dimer profile in heparin-treated patients. In terms of secondary outcomes, heparin therapy appeared to slow resolution of oxygenation indices in patients with COVID-19 ARDS. Patients receiving heparin therapy had lower PaO_2_/FiO_2_ ratios, increased oxygenation indices, and decreased ROX index profiles, up to day 10. The time to separation from respiratory support, and the time to ICU or hospital discharge was similar in both groups.

This study was an open-label study, with a small number of patients, which limits the inferences that can be made from the outcomes noted. The lack of any signal of harm in terms of safety outcomes may suggest that nebulised heparin is a safe treatment in this patient group. Of note, there were no bleeding complications noted which required blood transfusion, and there were no significant changes in systemic coagulation parameters. This would suggest that the intention of avoiding systemic absorption was achieved. The study also demonstrated nebulised heparin to be a well-tolerated treatment, both in invasively ventilated patients and awake patients.

In terms of biochemical markers of inflammation, results did not suggest any significant effect on the level of inflammation seen in COVID 19 patients requiring advanced respiratory support. This may be related to the small study numbers, lack of an actual effect, or to systemic inflammation not affected by the local administration of nebulised heparin. There was no demonstrable effect of the inhaled heparin on indices of coagulation, which provides reassurance that systemic absorption of the nebulised heparin was limited.

However, there were signs of detrimental effects on respiratory indices in the nebulised heparin group in this study, with patients having slower improvement in indices of oxygenation, compared to those that received standard care. Again, in view of the open label nature of the study, and small number of patients recruited, it is difficult to infer any definite conclusions from this. There were no between-group differences in pulmonary compliance, another marker of ARDS severity. However, this was only measurable in the invasive mechanical ventilation cohort within the study, so it is not possible to derive any definitive conclusions from this outcome. Reassuringly, there was no between-group difference in the time to separation from respiratory support, or in the time to ICU or hospital discharge. Nevertheless, the lack of any signal for benefit, in terms of clinical indices, or in indices of inflammation or coagulation means careful consideration would be required before proceeding to larger studies of this intervention in this patient cohort.

Our findings contrast with the results from a similar trial from Brazil, which reported a potential signal for benefit with inhaled unfractionated heparin [15]. In addition, a multi-centre international case series found that inhaled heparin therapy significantly improved oxygenation in both intubated and non-intubated patients [16]. The results from this trial will be included in ongoing meta-trials enrolling patients receiving invasive ventilation [17] and patients receiving other forms of advanced respiratory support [18]. It is hoped that these meta-trials will provide additional insights into the therapeutic potential of inhaled heparin for COVID-19-induced severe respiratory failure.

There are some limitations to be considered. Firstly, this is a small, early phase, open-label clinical trial, and so it is not possible to derive any definitive conclusions from this data. Second, this study was limited to patients with COVID-19 induced ARDS, and so should not be extrapolated to patient with ‘classic’ ARDS, where there is data to suggest therapeutic promise [1, 2]. Third, this was an open-label trial without a nebulised placebo in the standard care comparator group, as it was not considered justified to nebulise a placebo fluid in these patients. The effect of nebulised particles on the lung, without accounting for the heparin effect was therefore not accounted for in this small early phase study, which could be relevant. However, in a previous, bigger study by Dixon et al. the nebulisation of placebo did not produce measurable effects [2]. Fourth, we did not assess local inflammatory markers, such as those obtained via bronchoalveolar lavage, as these samples could only be generated when a clear clinical indication for this procedure existed.

Lastly, we used d-dimers as our surrogate efficacy endpoint, a choice based on emerging data at that time demonstrating a role for d-dimers in the pathophysiology of COVID-19, and in predicting outcomes from severe COVID-19 [3]. This paper reported a strong association between higher d-dimer concentrations and poorer outcomes in patients with severe COVID-19 requiring invasive ventilation. The paper further described a potential causal mechanism linking higher d-dimer concentrations with the development of areas of pulmonary hypoperfusion on CT pulmonary angiograms, consistent with thromboembolic disease, in these patients. While it is possible that treatments such as steroids and tocilizumab may alter d-dimer concentrations, the use of these therapies was not different between the groups in our study. Our findings are robust in terms of demonstrating no effect of nebulised heparin on d-dimer concentrations.

## Conclusions

In this phase Ib/IIa clinical trial, we found that nebulised unfractionated heparin was safe and well tolerated in patients with severe COVID-19 induced respiratory failure requiring advanced respiratory support. However, there was no evidence to suggest that nebulised heparin was effective. These findings indicate that careful consideration would be required before proceeding to larger studies of this intervention in this patient population.

## Supplementary Information

Supplementary Material 1: Figure e1: Evolution of Respiratory Indices from baseline to day 10 in patients receiving invasive mechanical ventilation. *Panel A:* Evolution of PaO2/FiO2 ratio in invasively ventilated patients receiving heparin treatment and SOC. *Panel B:* Evolution of ROX Index in invasively ventilated patients receiving heparin treatment and SOC. *Panel C:* Evolution of Oxygenation Index in invasively ventilated patients receiving heparin and SOC.

Supplementary Material 2: Table e1: Classification of Serious Adverse Events. Table e2: Detailed assessment of bleeding events reported as safety event. Table e3: Effect of first nebulised heparin dose on pre- versus 1hr post-delivery cardiovascular and respiratory indices. Table e4: Indices of coagulation over time.

## Data Availability

Investigators will consider all reasonable requests for access to study data on a case-by-case basis. Requests for data should be made by email to the corresponding author. Any data provided will consist of deidentified participant data, will be restricted to the data presented in this paper, and be subject to a data-sharing agreement.

## Abbreviations

SARS-COV-2: Severe acute respiratory syndrome coronavirus 2
ARDS: Acute respiratory distress syndrome
COVID 19: Coronavirus disease (COVID-19) is an infectious disease caused by the SARS-CoV-2 virus.
CT: Commuted tomography
SD: Standard deviation
PaO2/FiO2 ratio: Ratio of partial pressure of oxygen in arterial blood to the fraction of inspired oxygen.
PF ratio: Ratio of partial pressure of oxygen in arterial blood to the fraction of inspired oxygen.
ROX: Index ratio of oxygen saturation as measured by pulse oximetry/FIO2 to respiratory rate
ICU: Intensive Care Unit
EU: European Union
APTT: Activated partial thromboplastin time
IL: Interleukin
eCRF: Electronic Case Report Form
CRO: Contract Research Organisation
PRBCs: Packed red blood cells
HIT: Heparin induced thrombocytopaenia
SAE: Serious adverse event
AE: Adverse event
SOFA: Sequential Organ Failure Assessment
BMI: Body mass index
AUC: Area under the curve
CONSORT: Consolidated Standards of Reporting Trials
SOC: Standard of care
OI: Oxygenation index
RR: Relative risk
CI: Confidence interval
MV: Mechanical ventilation
TNF: Tumour necrosis
