# Supplementary material for: Can nebulised heparin reduce acute lung injury in patients with SARS‑CoV‑2 requiring advanced respiratory support in Ireland: the CHARTER‑Ireland phase Ib/IIa, randomised, parallel-group, open-label study
Source: Intensive Care Med Exp. 2025 Feb 7;13:15. doi: 10.1186/s40635-025-00727-x (PMC11806160; doi:10.1186/s40635-025-00727-x)
Supplement: Supplementary file 2 — Supplementary Material 2: Table e1: Classification of Serious Adverse Events. Table e2: Detailed assessment of bleeding events reported as safety event. Table e3: Effect of first nebulised heparin dose on pre- versus 1hr post-delivery cardiovascular and respiratory indices. Table e4: Indices of coagulation over time. [file 40635_2025_727_MOESM2_ESM.docx]

**Table e1: Classification of Serious Adverse Events**

| **Preferred Term** | **Heparin Treatment [N=20]** | **Standard Care [N=19]** | **p-value** |
| --- | --- | --- | --- |
| Covid-19 pneumonia n (%) | 2 (10%) | 2 (10.5%) | 1 |
| Oxygen saturation decreased n (%) | 1 (5%) | 1 (5.3%) | 1 |
| Abnormal loss of weight n (%) | 0 (0%) | 1 (5.3%) | 0.607 |
| Acute kidney injury n (%) | 0 (0%) | 1 (5.3%) | 0.607 |
| Alcohol withdrawal syndrome n (%) | 1 (5%) | 0 (0%) | 1 |
| Cardiac tamponade n (%) | 1 (5%) | 0 (0%) | 1 |
| Chest pain n (%) | 1 (5%) | 0 (0%) | 1 |
| Chronic obstructive pulmonary disease n (%) | 1 (5%) | 0 (0%) | 1 |
| Epistaxis n (%) | 0 (0%) | 1 (5.3%) | 0.607 |
| Hypoaesthesia n (%) | 0 (0%) | 1 (5.3%) | 0.607 |
| Ischaemic hepatitis n (%) | 1 (5%) | 0 (0%) | 1 |
| Liver function test increased n (%) | 1 (5%) | 0 (0%) | 1 |
| Peripheral artery occlusion n (%) | 0 (0%) | 1 (5.3%) | 0.607 |
| Pneumonia n (%) | 1 (5%) | 0 (0%) | 1 |
| Respiratory failure n (%) | 1 (5%) | 0 (0%) | 1 |
| Vocal cord paralysis n (%) | 0 (0%) | 1 (5.3%) | 0.607 |
| Vomiting n (%) | 0 (0%) | 1 (5.3%) | 0.607 |

**Table e2: Detailed assessment of bleeding events reported as a safety event**

| **Prefered Term** | **Heparin Treatment [N=20]** | **Standard Care [N=19]** | **p-value** |
| --- | --- | --- | --- |
| Epistaxis n (%) | 5 (25%) | 3 (15.8%) | 0.721 |
| Haematuria n (%) | 0 (0%) | 2 (10.5%) | 0.345 |
| Haemoptysis n (%) | 2 (10%) | 0 (0%) | 0.607 |
| Hypotension n (%) | 1 (5%) | 1 (5.3%) | 1 |
| Cardiac tamponade n (%) | 1 (5%) | 0 (0%) | 1 |
| Injection site haemorrhage n (%) | 1 (5%) | 0 (0%) | 1 |
| Peripheral artery occlusion n (%) | 0 (0%) | 1 (5.3%) | 0.607 |
| Post procedural haemorrhage n (%) | 0 (0%) | 1 (5.3%) | 0.607 |

**Table e3: Effect of first nebulized heparin dose on pre- versus 1hr post-delivery cardiovascular and respiratory indices**

|  | **Pre-Nebulization [N=20]** | **1Hr Post-Nebulization [N=19]** | **Paired comparison 95% CI (t.test or Wilcoxon test)** | **p-value** |
| --- | --- | --- | --- | --- |
| P/F Ratio; Mean (SD) (N 20,19) | 180.3 (64.3) | 165.4 (55.3) | Diff=-15.6 (CI -55.7 to 24.4) | 0.422 |
| Oxygenation Index; Median (IQR) (N 10,11) | 71.5 (378.6) | 88.6 (217.3) | Diff=-24.3 (CI -216.1 to 14.4) | 0.375 |
| ROX Index; Mean (SD) (N 20,19) | 9.2 (3) | 9.3 (2.8) | Diff=0.1 (CI -2 to 2.1) | 0.948 |
| Mean Arterial Blood Pressure; Mean (SD) (N 20,19) | 88.5 (15.2) | 84.3 (16.2) | Diff=-3.9 (CI -10 to 2.2) | 0.198 |
| Total SOFA Score; Median (IQR) (N 20,19) | 3 (5) | 5 (5.5) | Diff=1 (CI 0.5 to 2) | 0.012 |

**Table e4: Indices of coagulation over time**

|  | **Heparin Treatment [N=20]** | **Standard Care [N=19]** | **p-value** |
| --- | --- | --- | --- |
| **Activated Partial Thromboplastin Time (APTT) Mean (SD)** | | | |
| Baseline | 22.7 (3.6) | 23.4 (3.2) | 0.495 |
| Final (N 18,16) | 22.1 (3.4) | 24.5 (10.6) | 0.393 |
| Delta (Final - Baseline) (N 18,16) | -0.8 (3.3) | 0.7 (10.1) | 0.587 |
| **Maximum Activated Partial Thromboplastin Time (APTT) Median (IQR)** | | | |
| Overall | 23.2 (3) | 24.2 (6) | 0.565 |
| **International Normalised Ratio (INR) Median (IQR)** | | | |
| Baseline | 1 (0.1) | 1 (0) | 0.09 |
| Final (N 18,16) | 1 (0.1) | 1 (0.1) | 0.898 |
| Delta (Final - Baseline) (N 18,16) | 0 (0.1) | 0 (0.1) | 0.239 |
| **Platelet Count Median (IQR)** | | | |
| Baseline | 232.5 (114.8) | 287 (116.5) | 0.482 |
| Final (N 18,17) | 259.5 (149.5) | 248 (138) | 0.741 |
| Delta (Final- Baseline) (N 18,17) | 9.8 (184.5) | 9.2 (138.1) | 0.961 |
| **Prothrombin Time (PT) Median (IQR)** | | | |
| Baseline | 11.3 (0.9) | 10.9 (0.8) | 0.226 |
| Final (N 18,16) | 11.1 (0.6) | 11.2 (0.5) | 0.809 |
| Delta (Final- Baseline) (N 18,16) | -0.1 (0.9) | 0 (0.9) | 0.653 |
| **Fibrinogen Median (IQR)** | | | |
| Baseline (N 19,18) | 5.5 (2.8) | 4.6 (2.3) | 0.438 |
| Final (N 17,16) | 3.3 (2.4) | 2.9 (1.8) | 0.471 |
| Delta (Final- Baseline) (N 16,15) | -1.8 (2.3) | -1.8 (2.5) | 0.813 |
| **Haemoglobin (g/dL) Mean (SD)** | | | |
| Baseline | 13.3 (1.8) | 13.3 (1.4) | 0.889 |
| Final (N 19,17) | 12.7 (2.5) | 12.5 (2) | 0.799 |
| Delta (Final- Baseline) (N 19,17) | -0.8 (1.9) | -1 (1.8) | 0.736 |
